# Leisure-Time Physical Activity and Its Association With Metabolic Risk Factors in Iranian Adults: Tehran Lipid and Glucose Study, 2005–2008

**DOI:** 10.5888/pcd10.120194

**Published:** 2013-03-14

**Authors:** Bita Faam, Farhad Hosseinpanah, Atieh Amouzegar, Arash Ghanbarian, Golaleh Asghari, Fereidoun Azizi

**Affiliations:** Author Affiliations: Bita Faam, Atieh Amouzegar, Arash Ghanbarian, Golaleh Asghari, Fereidoun Azizi, Shahid Beheshti University of Medical Sciences, Tehran, Iran.

## Abstract

We examined the association between leisure-time physical activity (LTPA) and metabolic syndrome (MetS) among 4,665 randomly selected adults who participated in the Tehran Lipid and Glucose Study, 2005–2008. Normal-weight participants with light LTPA had higher risk of low high-density lipoprotein cholesterol and elevated levels of triglycerides than those with vigorous LTPA. Overweight adults with moderate LTPA had higher risk of having elevated levels of fasting blood glucose than adults with vigorous LTPA and, in the same group, we found an inverse association between light LTPA and MetS after adjustment for sex, age, education levels, smoking, and calorie intake. Although participants in the normal-weight and obese groups with vigorous LTPA had higher risk of high systolic blood pressure than participants with moderate LTPA, this finding had no clinical significance. Increased LTPA is associated with decreased risk of any damaging changes in the markers of MetS.

## Objective

Metabolic syndrome (MetS) is caused by a combination of an unhealthy diet, sedentary lifestyle, and genetic predisposition (1). Prevalence of MetS in South Asian populations is approximately 30%, depending on the region, duration and extent of urbanization, and lifestyle, and in Iranian populations is 32% (2). Recommendations for prevention and treatment of MetS and its components are engaging in physical activity, losing weight, and eating a healthful diet. Leisure-time physical activity (LTPA) plays an important role in controlling MetS (3,4). The aim of this study was to examine the association between LTPA and MetS among adults in the Tehran Lipid and Glucose Study (TLGS).

## Methods

Initially, 9,376 adults aged 20 to 70 years were selected randomly from among TLGS participants (5) for this cross-sectional study; after excluding participants who had diabetes mellitus (n = 390), body mass index (BMI) less than 18 kg/m^2^ (n = 152), or lacked complete data for variables (n = 4,169), 4,665 remained (1,976 men and 2,689 women). This study was approved by the institutional review board of the Research Institute for Endocrine Sciences, Shahid Beheshti University of Medical Sciences. Demographic and biochemical variables were measured (5), and people were classified on the basis of BMI levels. We defined MetS according to the Joint Interim Statement (JIS) guidelines issued by the International Diabetes Federation Task Force on Epidemiology and Prevention; National Heart, Lung, and Blood Institute; American Heart Association; World Heart Federation; International Atherosclerosis Society; and International Association for the Study of Obesity (6,7). We collected information about physical activity by using the Modifiable Activity Questionnaire (MAQ) (8) to calculate metabolic equivalent task (MET) minutes per week. High reliability (98%) and moderate validity (47%) were found for the MAQ translated into Persian (8,9). Levels of LTPA were categorized as light (MET <600 min/wk), moderate (MET 600–1,499 min/wk), and vigorous (MET ≥1,500 min/wk) (10). Dietary intake was assessed by using a 168-item validated semiquantitative food frequency questionnaire (FFQ) (11). Each food and beverage was analyzed for energy (kcal) content by using the US Department of Agriculture’s food composition database. Levels of education were categorized as elementary or secondary, high school, and bachelor’s degree or higher. Participants with current or past history of smoking were categorized as smokers and those who had never smoked were categorized as nonsmokers.

### Statistical analysis

We used the χ^2^ test to compare LTPA levels between BMI groups. Analysis of covariance was used to calculate the means of individual MetS components in various BMI groups. Multiple logistic regressions were performed to determine the association between individual MetS components and LTPA. All statistical analyses were performed by SPSS version 16.0 (IBM, Chicago, Illinois); tests were 2-sided, and difference was considered statistically significant at *P* < .05.

## Results

Mean age of participants was 40.7 (standard deviation [SD], 13.9) years. Of these participants, 1,108 (23.7%) were obese (BMI ≥30). According to the JIS definition, 31.5% of the 4,665 participants had MetS. Approximately 17% of participants had engaged in vigorous LTPA. The obese group had significantly higher weight, waist circumference, BMI, fasting blood glucose, systolic and diastolic blood pressure, and lower high-density lipoprotein (HDL) cholesterol than normal and overweight groups. The prevalence of MetS was significantly higher in the obese group (58.2%) than among overweight (36.6%) and normal-weight adults (6%). We found no significant difference between BMI groups regarding LTPA categories, which were proportionally distributed between groups (Table 1).

**Table 1:** Demographic and Metabolic Characteristics of Participants by Body Mass Index (BMI)^a^, the Tehran Lipid and Glucose Study, 2005–2008^b^

Variable	BMI 18–24.9 kg/m^2^ (n = 1,565)	BMI 25–29.9 kg/m^2^ (n = 1,992)	BMI ≥30 kg/m^2^ (n = 1,108)	Total (n = 4,665)
Age, y (SD)	34.8 (13.6)	42.7 (13.1)	45.3 (12.6)	40.7 (13.9)
No. of men (%)	716 (45.5)	915 (45.9)	345 (31.1)	1,976 (42.4)
**Smoker, n (%)**				
Current	172 (11.1)	226 (11.4)	99 (8.9)	497 (10.6)
Past	112 (7.2)	174 (8.7)	67 (6.1)	353 (7.6)
**Clinical measurement**				
No. with metabolic syndrome (%)	94 (6.0)	729 (36.6)	645 (58.2)^c^	1,468 (31.5)
Weight, kg (SD)	61.4 (8.3)	73.5 (9.2)	85.9 (12.7)^c^	72.4 (13.5)
Height, cm (SD)	165.2 (9.5)	163.6 (9.6)	160.3 (9.8)	163.3 (9.8)
Waist circumference, cm (SD)	78.1 (8.6)	90.8 (8.2)	102.1 (9.3)^c^	89.2 (12.4)
BMI, kg/m^2^ (SD)	22.4 (1.8)	27.4 (1.4)	33.3 (3.1)^c^	27.1 (4.6)
Fasting blood glucose, mg/dL (SD)	85.2 (1.1)	88.9 (1.1)	91.2 (1.1)^c^	88.2 (1.1)
Triglycerides, mg/dL (SD)	111.4 (1.6)	153.6 (1.6)	147.1 (1.6)^c^	143.7 (88.7)
High-density lipoprotein cholesterol, mg/dL (SD)	44.1 (10.5)	41.3 (9.7)	41.2 (9.9)^c^	42.2 (10.1)
Systolic blood pressure, mm Hg (SD)	106.9 (14.3)	113.8 (16.3)	119.1 (17.2)^c^	112.7 (16.6)
Diastolic blood pressure, mm Hg, (SD)	69.5 (8.9)	73.7 (9.6)	77.7 (9.9)^c^	73.2 (10.1)
**Education (%)**				
Elementary or secondary	256 (16.4)	596 (29.9)	486 (43.9)^c^	1,338 (28.7)
High school	827 (52.8)	875 (43.9)	423 (38.2)^c^	2,125 (45.6)
Bachelor’s degree or higher	452 (28.9)	458 (23.0)	145 (13.1)^c^	1,055 (22.6)
**Leisure-time physical activity (LTPA), n (%)**				
Light LTPA (MET <600 min/wk)	794 (50.7)	1,011 (50.8)	578 (52.2)	2,383 (51.1)
Moderate LTPA (MET 600–1,499 min/wk)	488 (31.2)	637 (32.0)	365 (32.9)	1,490 (31.9)
Vigorous LTPA (MET ≥1,500 min/wk)	283 (18.1)	344 (17.3)	165 (14.9)	792 (17.0)

Abbreviations: SD, standard deviation; MET, metabolic equivalent task.

a BMI is calculated as weight in kilograms divided by the square of height in meters (kg/m^2^).

b Data were log transformed. Values for all of the variables were adjusted for age and sex.

c Differences between groups were assessed by using analysis of covariance; significance was set at *P* < .05.

In the obese group, systolic blood pressure and triglyceride levels differed significantly by LTPA categories (*P* = .03 for systolic blood pressure, *P* = .01 for triglyceride level) (Figure). Normal-weight adults who participated in light LTPA had a higher risk of having elevated triglycerides and reduced HDL cholesterol than did adults who participated in vigorous LTPA (odds ratio [OR] for elevated triglycerides, 1.46; 95% confidence interval [CI], 1.01–2.14; *P* = .049) (OR for reduced HDL, 1.15; 95% CI, 1.05–2.33; *P* = .03) (Table 2). However, these associations were attenuated after adjustment for sex, age, smoking status, education levels, and calorie intake. In the overweight group, adults who participated in moderate LTPA had higher fasting blood glucose levels than those with vigorous LTPA after adjustment for sex, age, smoking status, education levels, and calorie intake (OR, 1.65; 95% CI, 1.37–3.23; *P* = .02), and the association between MetS and light LTPA was significant only after adjustment for the same variables (OR, 2.08; 95% CI, 1.03–4.21; *P* = .04). Normal-weight participants with vigorous LTPA had a greater risk of having high systolic blood pressure than did those whose LTPA was moderate (OR, 0.52; 95% CI, 0.31–0.86; *P* = .01), and the same risk was found in obese people who participated in vigorous LTPA compared with light LTPA (OR, 0.60; 95% CI, 0.41–0.91; *P* = .01) and moderate LTPA (OR: 0.58; 95% CI, 0.39–0.64; *P* = .005) (Table 2).

**Figure:**
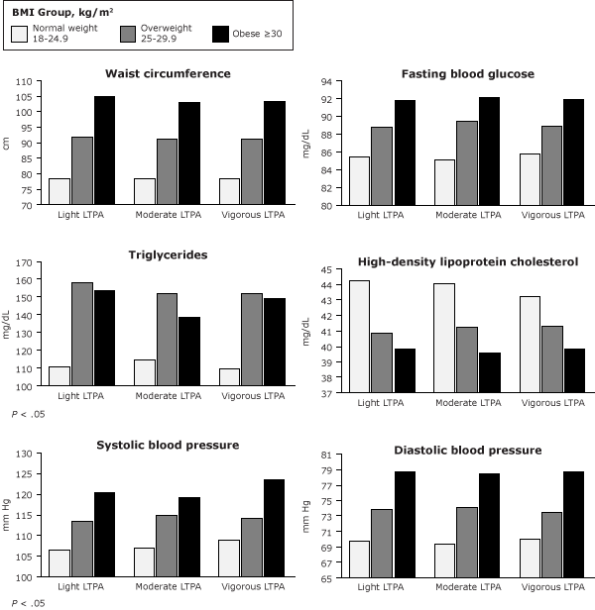
Systolic blood pressure and triglyceride levels differed significantly for obese adults depending on their LTPA level. Abbreviations: BMI, body mass index; LTPA, leisure-time physical activity.

**Table:** Waist circumference, cm

BMI Group, kg/m^2^	Light LTPA	Moderate LTPA	Vigorous LTPA
Normal weight, 18-24.9	78.2	78.2	78.2
Overweight, 25-29.9	91.3	91.0	90.9
Obese, ≥30	104.6	102.5	103.2

**Table:** Triglycerides, mg/dL

BMI Group, kg/m^2^	Light LTPA	Moderate LTPA	Vigorous LTPA
Normal weight, 18-24.9	111.1	114.9	109.6
Overweight, 25-29.9	153.5	138.4	149.2
Obese, ≥30	158.0	152.0	152.0

*P* < .05.

**Table:** Systolic blood pressure, mm Hg

BMI Group, kg/m^2^	Light LTPA	Moderate LTPA	Vigorous LTPA
Normal weight, 18-24.9 2	106.4	106.9	108.9
Overweight, 25-29.9	113.5	114.8	114.3
Obese, ≥30	120.4	119.2	123.3

*P* < .05.

**Table:** Fasting blood glucose, mg/dL

BMI Group, kg/m^2^	Light LTPA	Moderate LTPA	Vigorous LTPA
Normal weight, 18-24.9	85.5	85.1	85.8
Overweight, 25-29.9	88.8	89.5	88.9
Obese, ≥30	91.8	92.2	92.0

**Table:** High-density lipoprotein cholesterol, mg/dL

BMI Group, kg/m^2^	Light LTPA	Moderate LTPA	Vigorous LTPA
Normal weight, 18-24.9	44.2	44.0	43.2
Overweight, 25-29.9	40.8	41.2	41.3
Obese, ≥30	39.8	39.5	39.8

**Table:** Diastolic blood pressure, mm Hg

BMI Group, kg/m^2^	Light LTPA	Moderate LTPA	Vigorous LTPA
Normal weight, 18-24.9	69.7	69.3	70.0
Overweight, 25-29.9	73.9	74.1	73.4
Obese, ≥30	78.7	78.4	78.7

**Table 2:** Association Between Leisure-Time Physical Activity^a^ and Metabolic Syndrome Components, by Body Mass Index (BMI)^b^, Tehran Lipid and Glucose Study, 2005–2008^c^

Metabolic Syndrome Component	Crude Odds Ratio (95% CI)	*P* Value	Adjusted Odds Ratio (95% CI)	*P* Value
**BMI 18-24.9 kg/m^2^**				
**Abdominal obesity**				
Vigorous LTPA	1 [Reference]			
Moderate LTPA	1.29 (0.47-3.50)	.62	0.25 (0.03-2.11)	.21
Light LTPA	1.76 (0.63-4.90)	.28	1.42 (0.22-9.38)	.71
**Fasting blood glucose**				
Vigorous LTPA	1 [Reference]			
Moderate LTPA	1.02 (0.45-1.57)	.60	1.31 (0.14-2.31)	.81
Light LTPA	1.05 (0.52-1.93)	.98	1.52 (0.19-2.51)	.61
**Elevated triglycerides**				
Vigorous LTPA	1 [Reference]			
Moderate LTPA	1.12 (0.78-1.61)	.53	0.79 (0.37-1.68)	.54
Light LTPA	1.46 (1.01-2.14)	.049	1.01 (0.46-2.21)	.98
**Low HDL-C**				
Vigorous LTPA	1 [Reference]			
Moderate LTPA	0.76 (0.58-1.01)	.06	1.00 (0.55-1.74)	.95
Light LTPA	1.15 (1.05-2.33)	.03	0.77 (0.42-1.41	.41
**Elevated SBP**				
Vigorous LTPA	1 [Reference]			
Moderate LTPA	0.52 (0.31-0.86)	.01	1.02 (0.24-4.31)	.97
Light LTPA	0.69 (0.40-1.17)	.17	1.05 (0.22-4.48)	.99
**Elevated DBP**				
Vigorous LTPA	1 [Reference]			
Moderate LTPA	1.29 (0.63-2.65)	.48	1.76 (0.46-6.81)	.41
Light LTPA	1.29 (0.61-2.76)	.51	0.86 (0.19-3.92)	.85
**MetS**				
Vigorous LTPA	1 [Reference]			
Moderate LTPA	0.98 (0.51-1.59)	.73	1.41 (0.28-7.13)	.41
Light LTPA	0.96 (0.52-1.76)	.91	1.12 (0.19-6.37)	.12
**BMI 25-29.9 kg/m^2^**				
**Abdominal obesity**				
Vigorous LTPA	1 [Reference]			
Moderate LTPA	1.01 (0.66-1.10)	.23	1.01 (0.51-1.88)	.94
Light LTPA	1.03 (0.71-1.22)	.61	1.02 (0.45-1.76)	.73
**Fasting blood glucose**				
Vigorous LTPA	1 [Reference]			
Moderate LTPA	0.80 (0.54-1.19)	.27	1.65 (1.37-3.23)	.02
Light LTPA	1.01 (0.67-1.53)	.95	1.47 (1.28-3.31)	.06
**Elevated triglycerides**				
Vigorous LTPA	1 [Reference]			
Moderate LTPA	1.09 (0.85-1.41)	.47	1.06 (0.61-1.87)	.82
Light LTPA	1.01 (0.67-1.53)	.35	1.29 (0.72-2.34)	.39
**Low HDL-C**				
Vigorous LTPA	1 [Reference]			
Moderate LTPA	1.03 (0.75-1.23)	.75	1.24 (0.72-2.15)	.43
Light LTPA	1.04 (0.74-1.25)	.78	1.35 (0.76-2.41)	.31
**Elevated SBP**				
Vigorous LTPA	1 [Reference]			
Moderate LTPA	0.78 (0.56-1.08)	.14	0.74 (0.33-1.65)	.46
Light LTPA	1.00 (0.70-1.41)	.98	1.22 (0.54-2.77)	.64
**Elevated DBP**				
Vigorous LTPA	1 [Reference]			
Moderate LTPA	1.00 (0.56-1.16)	.25	1.23 (0.53-2.84)	.63
Light LTPA	1.01 (0.65-1.41)	.82	1.17 (0.49-2.81)	.72
**MetS**				
Vigorous LTPA	1 [Reference]			
Moderate LTPA	0.93 (0.72-1.20)	.59	1.87 (0.95-3.67)	.07
Light LTPA	1.07 (0.81-1.40)	.63	2.08 (1.03-4.21)	.04
**BMI ≥30 km/m^2^**				
**Abdominal obesity**				
Vigorous LTPA	1 [Reference]			
Moderate LTPA	1.42 (0.92-2.16)	.11	1.12 (0.35-3.59)	.85
Light LTPA	0.88 (0.57-1.36)	.58	0.75 (0.23-2.42)	.63
**Fasting blood glucose**				
Vigorous LTPA	1 [Reference]			
Moderate LTPA	0.69 (0.46-1.07)	.09	0.47 (0.18-1.22)	.12
Light LTPA	0.76 (0.49-1.98)	.24	0.79 (0.31-2.08)	.63
**Elevated triglycerides**				
Vigorous LTPA	1 [Reference]			
Moderate LTPA	1.12 (0.79-1.58)	.52	1.85 (0.81-4.22)	.14
Light LTPA	0.80 (0.55-1.16)	.24	1.39 (0.58-3.31)	.46
**Low HDL-C**				
Vigorous LTPA	1 [Reference]			
Moderate LTPA	0.77 (0.54-0.89)	.15	1.13 (0.51-2.51)	.77
Light LTPA	1.08 (0.63-1.19)	.68	1.57 (0.68-3.63)	.29
**Elevated SBP**				
Vigorous LTPA	1 [Reference]			
Moderate LTPA	0.58 (0.39-0.64)	.005	0.52 (0.21-1.33)	.17
Light LTPA	0.60 (0.41-0.91)	.01	0.55 (0.21-1.46)	.23
**Elevated DBP**				
Vigorous LTPA	1 [Reference]			
Moderate LTPA	0.79 (0.54-1.18)	.26	0.67 (0.28-1.58)	.36
Light LTPA	0.65 (0.43-1.00)	.053	0.41 (0.16-1.02)	.06
**MetS**				
Vigorous LTPA	1 [Reference]			
Moderate LTPA	0.92 (0.65-1.32)	.68	1.01 (0.43-2.21)	.96
Light LTPA	0.72 (0.49-1.04)	.08	1.03 (0.38-2.11)	.82

Abbreviations: CI, confidence interval; LTPA, leisure time physical activity; HDL-C, high density lipoprotein cholesterol; SBP, systolic blood pressure; DBP; diastolic blood pressure; MetS, metabolic syndrome.

a Light LTPA is metabolic Equivalent of Task (MET) <600 min/wk; moderate LTPA is MET 600–1,499 min/wk; and vigorous LTPA is MET ≥1,500 min/wk.

b BMI is calculated as weight in kilograms divided by the square of height in meters (kg/m^2^).

c Values were adjusted for sex, age, smoking status, education levels, and calorie intake.

## Discussion

Our results demonstrate that for the normal-weight group with a sedentary lifestyle (light LTPA), the risk of having higher triglyceride levels was 46% and lower HDL cholesterol was 15% higher than for those with vigorous LTPA. Overweight people with vigorous LTPA had lower fasting blood glucose than moderate LTPA groups. Overweight people with a sedentary lifestyle had a higher risk of MetS than those who participated in vigorous LTPA. Previous studies suggested that increasing levels of LTPA, in terms of duration or intensity, were linearly associated with reduced risk of MetS (12).

Despite these findings, the results of some studies show no association between physical activity and MetS (13). The lack of association could be related to the criteria used for MetS, age range, or limited number of participants. Like us, other researchers found that elevated levels of HDL cholesterol were associated with vigorous LTPA, and a sedentary lifestyle was positively associated with elevated levels of triglycerides and fasting blood glucose (14). Unexpectedly, the risk of having elevated systolic blood pressure in the normal-weight and obese groups with vigorous LTPA was nearly 60% higher than in the overweight group. The difference in systolic blood pressure (3 mm Hg) between LTPA groups has no clinical importance but could be caused by regular participation in moderate or vigorous physical activity (15). 

Our study had a few limitations. First, this study was cross-sectional, so causality cannot be determined. Second, self-reported LTPA did not provide accurate estimates of absolute amounts of activity.

Engaging in any LTPA was associated with lower prevalence of MetS. Future studies are needed to investigate the association between various kinds of physical activity and MetS by considering factors that affect lifestyle, such as socioeconomic status.
